# HDOP and VDOP Analysis in an Ideal Placement Environment for Dual GNSSs

**DOI:** 10.3390/s22093475

**Published:** 2022-05-03

**Authors:** JinHyeok Jang, Dana Park, Sangkyung Sung, Young Jae Lee

**Affiliations:** Mechanical and Aerospace Engineering, Konkuk University, Seoul 05029, Korea; bassjang12@konkuk.ac.kr (J.J.); pdn0113@konkuk.ac.kr (D.P.); sksung@konkuk.ac.kr (S.S.)

**Keywords:** global navigation satellite system (GNSS), dual GNSS, horizontal dilution of precision (HDOP), vertical dilution of precision (VDOP)

## Abstract

Increasing the number of satellites in a global navigation satellite system (GNSS) improves the positioning accuracy and increases availability. However, it reduces the positioning accuracy improvement rate and increases the calculation loads, which can cause battery usage problems in mobile devices using a GNSS. An appropriate satellite selection method is required. One current method entails the use of ideal satellite placement with respect to the minimum geometric dilution of precision (GDOP). In this study, the described ideal satellite placement with the minimum GDOP were divided in terms of the horizontal dilution of precision (HDOP) and vertical dilution of precision (VDOP). HDOP and VDOP were mathematically derived and analyzed. The derived formula was verified using simulations. The analysis was performed with actual dual GNSS satellite data. The satellites adjacent to the ideal placement were selected and the DOP was calculated. Simply selecting satellites closest to the ideal placement afforded large values for HDOP and VDOP. This issue was addressed using a satellite changing algorithm considering the dual GNSS, resulting in reduced values of the HDOP and VDOP.

## 1. Introduction

As global navigation satellite systems (GNSSs) have become more popular and important, an increasing number of countries are developing and operating their own GNSSs. Typical examples include the United States’ global positioning system (GPS), Russia’s global navigation satellite system (GLONASS), the European Union’s Galileo, and China’s BeiDou. In addition to GNSSs, whose service areas encompass the entire globe, countries are also developing and operating regional navigation satellite systems (RNSSs), in which only certain regions are the serviced areas. Examples of RNSSs include India’s navigation with Indian constellation (NavIC) and Japan’s Quasi-Zenith satellite system (QZSS). Furthermore, South Korea is also promoting projects related to an RNSS, known as the Korea positioning system (KPS) [1]. In this manner, the number of navigation satellites has been increasing rapidly owing to the increase in navigation satellite systems [2]. As of January 2022, the number of GNSS satellites in operation were 31 in GPS, 21 in GLONASS, 22 in Galileo, and 44 in BeiDou [3,4,5,6]. Over time, the number of navigation satellites is expected to increase further.

As the number of navigation satellites increases, the number of visible satellites that can be used for positioning estimations also increases. Intuitively, it can be expected that the positioning accuracy will improve as the number of measurement values used in the positioning estimations are augmented. In addition, availability increases as positioning estimations become possible in environments with poor satellite visibility, such as urban and mountainous regions [7]. However, there are also drawbacks to this increase in the number of visible satellites. As the number of satellites used in positioning estimations increases, the computational load also increases. This, in turn, causes an increase in electricity consumption. These increments in electricity consumption can constitute a severe problem for mobile devices such as smartphones, which currently use GNSSs on a large scale [8]. In addition, the extent of accuracy improvements decreases as the number of visible satellites increases. This phenomenon can be confirmed based on the fact that the minimum geometric dilution of precision (GDOP) is expressed as a fractional function of the number of satellites [8]. Consequently, studies on satellite selection have been conducted.

For instance, studies have focused on determining the satellite with the minimum GDOP among the subsets of all satellites, by using iterative methods in a GPS environment [9]. In the case of the GDOP, which is the main index used for satellite selection, the calculation load increases with the number of visible satellites increases. Various studies have also been conducted to compensate for this problem [10,11]. These studies were effective in reducing the GDOP calculations when selecting satellites. However, the rapidly increasing number of navigation satellites is causing an exponential increase in the number of satellite subsets that must be calculated. This may cause difficulties when employing the methods adopted in previous studies. The GDOP is related to the volume of the polyhedron formed by a user and the satellites. Accordingly, studies have focused on developing suitable techniques to select satellites based on this concept [12,13,14]. However, in these studies, there were difficulties pertaining to the composition of the polyhedron and the volume calculations when the number of selected satellites increased. Research has also been performed on successively reducing the number of satellites by using the position dilution of precision (PDOP) of all of the satellites and the amount of change in the PDOP when one visible satellite is removed [15]. As the satellites are removed one at a time, a minimum value of the PDOP can be maintained during this process. However, a global minimum cannot be guaranteed since the results are obtained sequentially. Studies have also been conducted on empirically determining the placement with the minimum GDOP and selecting satellites that are close to these positions [8,16].

This study focused on the following content. The ideal satellite placement for minimizing the GDOP obtained in a previous study [16] is described. This method places satellites at the zenith and horizon, where the horizon satellites are spaced evenly. This placement approach is termed as the Zenith + Horizon (ZH) method. This study employed the satellite placement conditions under the ZH method without modifications, in order to derive the dilution of precision (DOP) mathematically. This mathematic derivation was performed to analytically interpret the information that was obtained empirically.

Each GNSS provides services on its own reference time [17]. When estimating the position in a multiple GNSSs, it is necessary to handle the individual GNSS’ reference time and receiver hardware delay. There are two methods for resolving this problem [18]. The first method involves calculating the position via the unknown values of one receiver’s clock error by correcting the reference time between the GNSSs, with the information obtained from the broadcast ephemeris. This method cannot compensate for the receiver hardware delay. The second method entails estimating the position by adding the receiver’s clock error as an unknown value depending on the number of receiving GNSSs. Unlike the first method, the second method can compensate for the receiver hardware delay. Although the first method uses multiple GNSSs, the DOP calculation is the same as that of a single GNSS. Since this study is conducted in a single GNSS, the first method is also studied. Which one of these two methods has better position accuracy depends on the environment; therefore, neither method can be considered superior [18]. For this reason, this study was performed using the second method.

Owing to the mathematical complexity associated with multiple GNSSs, a dual GNSSs was considered. It is expected that the results of studying a dual GNSS can be extended to multiple GNSSs. Furthermore, most existing studies were conducted considering the GDOP. This study employed the horizontal dilution of precision (HDOP) and the vertical dilution of precision (VDOP), instead of using the GDOP. With regard to a GNSS, the importance of the horizontal and vertical precision varies depending on the field of application. For instance, in the case of cars and boats, the horizontal accuracy is more important, whereas the vertical accuracy is more important for aircraft. The GDOP includes the 3D positions and clock errors. Hence, it expresses the average accuracy of the estimate. Therefore, depending on the field of use, it can be more effective to use the HDOP and VDOP as references, rather than the GDOP.

The contribution of this study can be summarized as follows:

1. Mathematical analysis of the simulated minimum DOP placement in a Dual GNSS;
2. Separation of GDOP into HDOP and VDOP to propose utilization according to application fields;
3. Using actual satellite data to verify formulas and propose possibilities using the satellite selection method.

The remainder of this study is organized as follows. Section 2 provides a brief description of the previously proposed ZH method [16]. In Section 3 and Section 4, the HDOP and VDOP are mathematically derived from a single GNSS and a dual GNSS’ ZH satellite placement. In Section 5, a ZH placement simulation considering a dual GNSS is performed based on the derived formulas, and the satellite placement is verified to ensure low values for the HDOP and VDOP. In Section 6, actual satellite data are used to confirm the effect of the proposed placement. Lastly, in Section 7, the content of this study is summarized, and the conclusions are presented.

## 2. Zenith + Horizon Method

The ZH method places a suitable number of satellites on the zenith and horizon for the minimum GDOP and selects the actual satellites located near these positions. Here, the placement conditions of the ZH method are first described. For the satellites that are placed at the zenith, duplication is possible. At the horizon, the satellites are placed in such a manner that the azimuth included angles between the satellites are identical. This placement is termed as the ZH placement.

**Figure 1:**
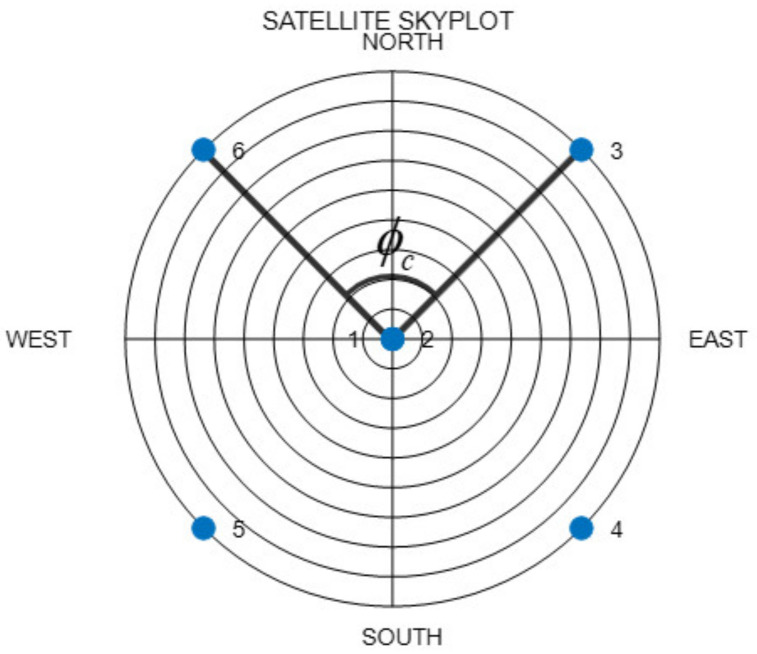
Example of satellite placement under the Zenith + Horizon (ZH) method.

Figure 1 shows an example of the ZH placement. The azimuth included angle between two satellites placed on the horizon can be expressed as in Equation (1):

(1) $$\phi_{c}=360{}^{\circ}/n_{u}.$$

Here,

$\phi_{c}$: Azimuth Included Angle

$n_{u}$: Number of Uniformly Placed Satellites

To ensures the minimum GDOP, the satellites placed on the horizon must all maintain elevation angles of 0°. In the subsequent discussions, we only consider that the elevation angles of the satellites placed on the horizon are identical, without the need for these angles to be zero. Thus, these satellites can be depicted as uniform satellites.

In a previous study [16], the minimum GDOP was obtained when the ratio of the numbers of zenith satellites to the number of uniform satellites was 1:2. Figure 1 depicts the placement with the minimum GDOP, for a total of six satellites (two satellites placed at the zenith and four satellites placed uniformly). The actual satellite selection criteria are as follows: For the zenith satellites, the satellites with the highest elevation angles are selected from among the visible satellites. For the uniform satellites, the ZH method entails the calculation of as many regular polyhedron vertex vectors as necessary, based on the satellite with the lowest elevation angle. These vectors and their dot product are then used to select the closest satellites. Lastly, the results are checked for duplicated satellites. If there are duplicate satellites, the dot product is used to change the satellite selection.

**Figure 2:**
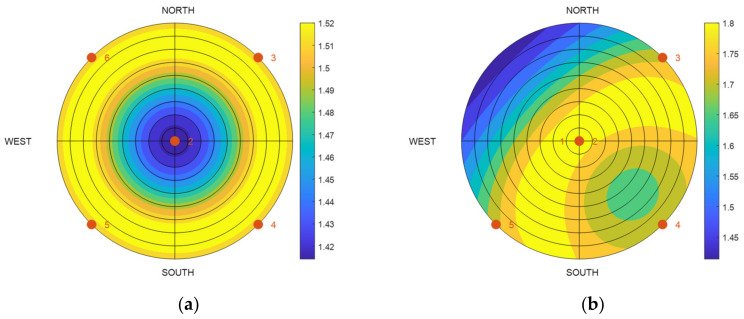
GDOP simulation according to the change in the position of one satellite in the ZH placement (six selected satellites): (**a**) satellite #1 is moved; (**b**) satellite #6 is moved.

When actual satellites are selected, the ZH placement cannot be maintained. However, even when the satellites are placed near the ZH placement, there are no problems with selecting the nearby positions, provided the DOP does not increase by a large amount. Figure 2 shows the GDOP when only one of the satellites in the placement in Figure 1 is moved with respect to the overall elevation angle and the azimuth. In Figure 2a, Satellite 1, which is placed at the zenith, is moved, whereas in Figure 2b, the uniform Satellite 6 is moved. In both these cases, it can be observed that the increase in the GDOP becomes larger as the satellite moves farther away from the position at which it was previously located. Although it varies according to the fixed satellites, selecting satellites that are close to the ZH placement allows for a lower DOP, in comparison to those at the other positions.

This study involved three changes to method adopted in a previous research [16]:

1. In this work, HDOP and VDOP were used instead of the GDOP used in the previous study;
2. Instead of four unknown values (three position coordinates + one single GNSS receiver clock), this study employed five unknown values (three position coordinates + two dual GNSS receiver clocks);
3. The previous results derived via simulations were analyzed via mathematical derivations.

## 3. Single GNSS Formula Derivation

The HDOP and VDOP were derived mathematically considering a state where the ZH placement conditions were maintained in a single GNSS. The derived results were then compared with the placement obtained using the GDOP [16], and the differences were subsequently analyzed.

Before deriving the formulas, the order of the satellite numbers was expressed using the following formula:

1. Zenith satellite 🡪 uniform satellite;
2. The uniform satellites were ordered from the lowest azimuth angle (from the north) to the highest.

The formulae used for the calculation of the DOP are expressed in Equations (2) and (3) [19].

(2) $$\begin{array}{cc} Q & ={\left(H^{T}H\right)}^{-1}\\ & =\left[\begin{array}{cccc} q_{EE} & q_{EN} & q_{EU} & q_{EC}\\ & q_{NN} & q_{NU} & q_{NC}\\ & & q_{UU} & q_{UC}\\ & & & q_{CC} \end{array}\right]. \end{array}$$

Here,

H: Observation matrix in ENU coordination

Q: Local cofactor matrix

q: Elements of local cofactor matrix

In Equation (2), Q is a symmetric matrix. In a symmetric matrix, only a triangle of the matrix elements is shown, whereas the symmetric terms are omitted.

(3) $$\begin{array}{l} HDOP=\sqrt{q_{EE}+q_{NN}}\\ VDOP=\sqrt{q_{UU}} \end{array}$$

In Equation (3), the elements of matrix Q are used to determine the HDOP and VDOP. For the ZH placement conditions, the HDOP and VDOP are calculated using the following process:

(4) $$\begin{array}{cc} H & =\left[\begin{array}{cccc} -\cos\theta^{1}\sin\phi^{1} & -\cos\theta^{1}\cos\phi^{1} & -\sin\theta^{1} & 1\\ \vdots & \vdots & \vdots & \vdots \\ -\cos\theta^{n}\sin\phi^{n} & -\cos\theta^{n}\cos\phi^{n} & -\sin\theta^{n} & 1 \end{array}\right]\\ & =\left[\begin{array}{cccc} 0 & 0 & -1 & 1\\ \vdots & \vdots & \vdots & \vdots \\ 0 & 0 & -1 & 1\\ -\cos\theta \sin\phi^{1_{u}} & -\cos\theta \cos\phi^{1_{u}} & -\sin\theta & 1\\ \vdots & \vdots & \vdots & \vdots \\ -\cos\theta \sin\phi^{n_{u}} & -\cos\theta \cos\phi^{n_{u}} & -\sin\theta & 1 \end{array}\right]. \end{array}$$

Here,

θ: Elevation angle (°)

ϕ: Azimuth angle (°)

${}^{n}$: Satellite number

Equation (4) presents the observation matrix used in the least squares estimation method, and it consists of the unit vectors between the user and the satellites, as well as terms related to the receiver’s clock error. Since the zenith satellite has an elevation angle of 90°, columns 1 and 2 are 0, whereas column 3 is −1. Since the uniform satellites all have the same elevation angles, they are expressed uniformly.

(5) $$\begin{array}{ll} H^{T}H & =\left[\begin{array}{cccc} \cos^{2}\theta \sum_{k=1}^{n_{u}}\sin^{2}\phi^{k} & \cos^{2}\theta \sum_{k=1}^{n_{u}}\frac{\sin2\phi^{k}}{2} & \frac{\sin2\theta }{2}\sum_{k=1}^{n_{u}}\sin\phi^{k} & -\cos\theta \sum_{k=1}^{n_{u}}\sin\phi^{k}\\ & \cos^{2}\theta \sum_{k=1}^{n_{u}}\cos^{2}\phi^{k} & \frac{\sin2\theta }{2}\sum_{k=1}^{n_{u}}\cos\phi^{k} & -\cos\theta \sum_{k=1}^{n_{u}}\cos\phi^{k}\\ & & n_{z}+n_{u}\sin^{2}\theta & -n_{z}-n_{u}\sin\theta \\ & & & n \end{array}\right]\\ \; & =\left[\begin{array}{cccc} \cos^{2}\theta \sum_{k=1}^{n_{u}}\sin^{2}\phi^{k} & 0 & 0 & 0\\ & \cos^{2}\theta \sum_{k=1}^{n_{u}}\cos^{2}\phi^{k} & 0 & 0\\ & & n_{z}+n_{u}\sin^{2}\theta & -n_{z}-n_{u}\sin\theta \\ & & & n \end{array}\right]. \end{array}$$

Here,

$n_{z}$: Number of zenith satellites

In Equation (5), if the condition of the uniform satellites is maintained and the number of satellites is 3 or more, the matrix elements (1,2), (1,3), (1,4), (2,3), and (2,4) are 0. To calculate the Q matrix, the inverse matrix of Equation (5) is used. Equation (5) can be expressed as a block matrix, as shown in Equation (6).

(6) $$H^{T}H=\left[\begin{array}{cc} B_{2\times 2}^{EN} & 0_{2\times 2}\\ & B_{2\times 2}^{UC} \end{array}\right].$$

Here,

B: Block matrix

0: Zero matrix

${}^{x}$: Coordinate of block matrix

${}_{x}$: Matrix size

In Equation (6), the HDOP can be calculated via the $B_{2\times 2}^{EN}$ inverse matrix, and the VDOP can be calculated via the $B_{2\times 2}^{UC}$ inverse matrix. The calculated HDOP and VDOP are shown in Equations (7) and (8):

(7) $$HDOP=\frac{1}{\sqrt{n_{u}}}\frac{2}{\cos\theta },$$

(8) $$VDOP=\sqrt{\frac{n}{n_{z}n_{u}}}\frac{1}{1-\sin\theta }.$$

Furthermore, Equations (7) and (8) can be divided into two parts. The first part is related to the number of satellites that are placed at the zenith and placed uniformly. The second part is related to the elevation angles of the uniform satellites. Looking at the first part, the HDOP decreases as the number of uniform satellites increases. If the number of selected satellites remains fixed, the VDOP decreases as the numbers of zenith and uniform satellites become nearly equal. In the second part, the DOP decreases as the elevation angles of the uniform satellites decrease. This indicates that the DOP is low if uniform satellites exist on the horizon, as reported previously [16]. Low-elevation satellites increase pseudorange errors [20]. This can reduce the positioning accuracy. To improve the positioning accuracy, additional studies on the elevation angle of the uniform satellites are required. In this study, only DOP is analyzed, so this content is not considered.

Table 1 presents the GDOP, HDOP, and VDOP results according to the numbers of zenith and uniform satellites, considering four to eight selected satellites. As mentioned earlier, three or more satellites are placed uniformly. In addition, when a zenith satellite is not placed, the calculation becomes impossible; therefore, a minimum of one satellite must be placed at the zenith. As can be observed, the GDOP decreases as the ratio of the number of zenith satellites to the number of uniform satellites approaches 1:2, as in previous studies [8,16]. Moreover, the HDOP decreases as the number of uniform satellites increases, as indicated by Equation (7). The VDOP also decreases as the numbers of zenith and uniform satellites become equal, as indicated by Equation (8).

## 4. Dual GNSS Formula Derivation

As in the equations for determining the HDOP and VDOP for a single GNSS, a mathematical derivation was performed using a dual GNSS.

(9) $$H=\left[\begin{array}{ccccc} -\cos\theta^{1_{z}^{a}}\sin\phi^{1_{z}^{a}} & -\cos\theta^{1_{z}^{a}}\cos\phi^{1_{z}^{a}} & -\sin\theta^{1_{z}^{a}} & 1 & 0\\ \vdots & \vdots & \vdots & \vdots & \vdots \\ -\cos\theta^{1_{z}^{b}}\sin\phi^{1_{z}^{b}} & -\cos\theta^{1_{z}^{b}}\cos\phi^{1_{z}^{b}} & -\sin\theta^{1_{z}^{b}} & 0 & 1\\ \vdots & \vdots & \vdots & \vdots & \vdots \\ -\cos\theta^{1_{u}^{a}}\sin\phi^{1_{u}^{a}} & -\cos\theta^{1_{u}^{a}}\cos\phi^{1_{u}^{a}} & -\sin\theta^{1_{u}^{a}} & 1 & 0\\ \vdots & \vdots & \vdots & \vdots & \vdots \\ -\cos\theta^{1_{u}^{b}}\sin\phi^{1_{u}^{b}} & -\cos\theta^{1_{u}^{b}}\cos\phi^{1_{u}^{b}} & -\sin\theta^{1_{u}^{b}} & 0 & 1\\ \vdots & \vdots & \vdots & \vdots & \vdots \end{array}\right]=\left[\begin{array}{ccccc} 0 & 0 & -1 & 1 & 0\\ \vdots & \vdots & \vdots & \vdots & \vdots \\ 0 & 0 & -1 & 0 & 1\\ \vdots & \vdots & \vdots & \vdots & \vdots \\ -\cos\theta \sin\phi^{1_{u}^{a}} & -\cos\theta \cos\phi^{1_{u}^{a}} & -\sin\theta & 1 & 0\\ \vdots & \vdots & \vdots & \vdots & \vdots \\ -\cos\theta \sin\phi^{1_{u}^{b}} & -\cos\theta \cos\phi^{1_{u}^{b}} & -\sin\theta & 0 & 1\\ \vdots & \vdots & \vdots & \vdots & \vdots \end{array}\right].$$

Here,

a: First GNSS

b: Second GNSS

The equation is expanded to a dual GNSS, and a fifth column is added to Equation (4). In the case of the second GNSS, the fifth column has a value of 1 [11].

(10) $$H^{T}H=\left[\begin{array}{ccccc} \cos^{2}\theta \sum_{k=1}^{n_{u}}\sin^{2}\phi^{k} & 0 & 0 & -\cos\theta \sum_{k=1}^{n_{u}^{a}}\phi^{k} & -\cos\theta \sum_{k=1}^{n_{u}^{b}}\sin\phi^{k}\\ & \cos^{2}\theta \sum_{k=1}^{n_{u}}\cos^{2}\phi^{k} & 0 & -\cos\theta \sum_{k=1}^{n_{u}^{a}}\cos\phi^{k} & -\cos\theta \sum_{k=1}^{n_{u}^{b}}\cos\phi^{k}\\ & & n_{z}+n_{u}\sin^{2}\theta & -n_{z}^{a}-n_{u}^{a}\sin\theta & -n_{z}^{b}-n_{u}^{b}\sin\theta \\ & & & n^{a} & 0\\ & & & & n^{b} \end{array}\right].$$

Equation (10) is comparable to Equation (5), which involved a similar process for the single GNSS. If uniformity is maintained in Equation (5), most of the elements of the nondiagonal matrix would become 0. In a dual GNSS, the matrix elements (1,4), (1,5), (2,4), and (2,5) are not 0. Therefore, it is difficult to perform a simple inverse matrix calculation, as in the case of a single GNSS. Equation (10) can also be expressed in the block matrix form, and the results are shown in Equation (11):

(11) $$H^{T}H=\left[\begin{array}{cc} B_{3\times 3}^{ENU} & B_{3\times 2}^{PC}\\ & B_{2\times 2}^{C} \end{array}\right].$$

In Equation (6), the horizon-related elements and the vertical or clock-related parts were separated. For a dual GNSS, the position-related term ($B_{3\times 3}^{ENU}$) and the clock-related term ($B_{2\times 2}^{C}$) are separated, as in Equation (11). Moreover, an additional term ($B_{3\times 2}^{PC}$) is present since the nondiagonal matrix is not 0. If the inverse matrix of Equation (11) is calculated, the result would be as shown in Equation (12):

(12) $${\left(H^{T}H\right)}^{-1}=\left[\begin{array}{cc} {\left(B_{3\times 3}^{ENU}-B_{3\times 2}^{PC}{\left(B_{2\times 2}^{C}\right)}^{-1}{\left(B_{3\times 2}^{PC}\right)}^{T}\right)}^{-1} & \\ & {\left(B_{2\times 2}^{C}-{\left(B_{3\times 2}^{PC}\right)}^{T}{\left(B_{3\times 3}^{ENU}\right)}^{-1}B_{3\times 2}^{PC}\right)}^{-1} \end{array}\right].$$

In this study, only the HDOP and VDOP are used; therefore, in Equation (12), only the (1,1) block matrix is considered. Since the calculation process is complex, it is not described in this Section. A detailed formula derivation is shown in Appendix A. Ultimately, when a dual GNSS is used, the HDOP and VDOP are as expressed in Equations (13) and (14):

(13) $$HDOP=\sqrt{\frac{1}{n_{u}}\frac{\left(n_{z}^{a}n_{u}^{a}n_{u}^{b}+n_{z}^{a}n_{u}^{a}n_{z}^{b}+n_{u}^{a}n_{z}^{b}n_{u}^{b}+n_{z}^{a}n_{z}^{b}n_{u}^{b}\right)-n_{z}SST}{\left(n_{z}^{a}n_{u}^{a}n_{u}^{b}+n_{z}^{a}n_{u}^{a}n_{z}^{b}+n_{u}^{a}n_{z}^{b}n_{u}^{b}+n_{z}^{a}n_{z}^{b}n_{u}^{b}\right)-2n_{z}SST}}\frac{2}{\cos\theta },$$

(14) $$VDOP=\sqrt{\frac{n^{a}n^{b}-\frac{2n}{n_{u}}SST}{\left(n_{z}^{a}n_{u}^{a}n_{u}^{b}+n_{z}^{a}n_{u}^{a}n_{z}^{b}+n_{u}^{a}n_{z}^{b}n_{u}^{b}+n_{z}^{a}n_{z}^{b}n_{u}^{b}\right)-2n_{z}SST}}\frac{1}{1-\sin\theta }.$$

In Equations (13) and (14), the square sum trigonometrical function (SST) is given by Equation (15):

(15) $$SST={\left(\sum_{k=1}^{n_{u}^{a}}\sin\phi_{k}\right)}^{2}+{\left(\sum_{k=1}^{n_{u}^{a}}\cos\phi_{k}\right)}^{2}={\left(\sum_{k=1}^{n_{u}^{b}}\sin\phi_{k}\right)}^{2}+{\left(\sum_{k=1}^{n_{u}^{b}}\cos\phi_{k}\right)}^{2}.$$

Equation (15) yields trigonometric functions with all of the azimuth angles of the uniform satellites for one of the dual GNSSs, and the squares of their sums are added. This value remains the same, regardless of which GNSS is considered. The properties of this value are explained in subsequent sections.

Here, the properties of the HDOP and VDOP in a dual GNSS are analyzed by considering Equations (13) and (14). As in the case of the single GNSS, both equations are separated into two parts, where one is related to the number of satellites and the other is related to the elevation angles of the uniform satellites. The analysis of the part related to the elevation angles is similar to that in the case of the single GNSS. Therefore, the description of this part is omitted; only the part related to the number of satellites in the square root is analyzed.

In Equation (13), this part in the square root is further divided into two parts: the first part only consists of $n_{u}$, and the second part has a form in which the numerator and denominator are similar but the subtracted term on the right side is doubled in the denominator. First, the correlation between $n_{u}$ and HDOP can be determined via the first part. In $n_{u}$, as the number of uniform satellites increases, the HDOP decreases. In this study, we analyzed the relationship between the HDOP and the variables included in the second part, namely $n_{z}^{a}n_{u}^{a}n_{u}^{b}+n_{z}^{a}n_{u}^{a}n_{z}^{b}+n_{u}^{a}n_{z}^{b}n_{u}^{b}+n_{z}^{a}n_{z}^{b}n_{u}^{b}$, $n_{z}$, and SST. All of these variables have positive values. $n_{z}^{a}n_{u}^{a}n_{u}^{b}+n_{z}^{a}n_{u}^{a}n_{z}^{b}+n_{u}^{a}n_{z}^{b}n_{u}^{b}+n_{z}^{a}n_{z}^{b}n_{u}^{b}$ is calculated via the satellites that are classified as zenith, uniform, first GNSS, and second GNSS. As this term increases, the values of the numerator and denominator become similar, and the HDOP decreases. $n_{z}$ is the number of zenith satellites, and SST is described briefly above. These two variables exhibit tendencies similar to that of the HDOP. As the two variables increase, the value subtracted from the denominator increases, and the HDOP consequently increases.

In Equation (14), for determining VDOP, n, $n^{a}n^{b}$, $n_{z}$, $n_{u}$, $n_{z}^{a}n_{u}^{a}n_{u}^{b}+n_{z}^{a}n_{u}^{a}n_{z}^{b}+n_{u}^{a}n_{z}^{b}n_{u}^{b}+n_{z}^{a}n_{z}^{b}n_{u}^{b}$, and SST are considered as variables. First, n is the number of selected satellites; as it increases, the VDOP decreases. However, for a more convenient analysis, this variable is considered as a fixed value. $n^{a}n^{b}$ is calculated from the number of satellites in each GNSS. As the numbers of satellites in each part become equal, its value increases, and the VDOP also increases. If $n_{z}$ increases, the denominator decreases, and the VDOP increases. If $n_{u}$ increases, the value of the numerator increases, and the VDOP increases. Furthermore, if $n_{z}^{a}n_{u}^{a}n_{u}^{b}+n_{z}^{a}n_{u}^{a}n_{z}^{b}+n_{u}^{a}n_{z}^{b}n_{u}^{b}+n_{z}^{a}n_{z}^{b}n_{u}^{b}$ increases, the denominator increases, and the VDOP decreases. The effect of the SST is dependent on the values of the other variables in the numerator and denominator; therefore, it does not have a tendency that is consistent with respect to the VDOP. Thus far, the individual effects of each of the variables that constitute Equation (14) have been examined. However, since these variables are not independent of each other, it is difficult to clearly discern the tendencies of the VDOP, as realized in the case of the HDOP. For example, if both $n_{z}$ and $n_{u}$ increase, the VDOP increases, as explained previously. However, these two values are related to $n=n_{z}+n_{u}$. Therefore, the two values cannot increase in the same manner. In addition, terms other than the SST are related to the number of satellites, and they are also correlated with each other. For these reasons, it is difficult to describe the effect of the variables on the VDOP through mathematic results alone. Therefore, the VDOP needs to be determined through simulations.

## 5. Simulation of Zenith + Horizon Placement Considering Dual GNSS

The HDOP and VDOP for the ZH placement considering a scenario with a dual GNSS were derived in Section 4. To verify the mathematically derived content, a simulation was performed. As the number of selected satellites increases, the number of simulations also increases; therefore, the number of satellites was limited to 6 in this study.

### 5.1. Horizontal Dilution of Precision

Using the derived formulas, it was confirmed that the HDOP declines if the number of uniform satellites increases, for both the single and dual GNSSs. However, if the selected satellites are all placed at a uniform satellite, the angles of elevation are identical. Therefore, the H matrix cannot have a full rank, and the position and DOP calculations become impossible. Therefore, one satellite is placed at the zenith to resolve this problem.

Table 2 presents the result of the dual GNSS HDOP simulation with one zenith satellite and five uniform satellites. All of the possible cases were simulated, and the HDOP values were arranged in ascending order. The $n_{z}^{a}n_{u}^{a}n_{u}^{b}+n_{z}^{a}n_{u}^{a}n_{z}^{b}+n_{u}^{a}n_{z}^{b}n_{u}^{b}+n_{z}^{a}n_{z}^{b}n_{u}^{b}$ variable in Equation (13) is represented as nC in Table 2. The final row in Table 2 denotes the case where the DOP calculations were impossible. In this case, the types of GNSSs used for the uniform satellites were the same, whereas those for the zenith satellite was different. Here, as in the case of placing uniform satellites alone, the H matrix cannot have a full rank, and the DOP calculations become impossible. In the simulation results, the minimum HDOP was calculated for the case involving a single GNSS. As can be observed, this is similar to the result of a previous study [21], where the DOP for a single GNSS was lower than that for a dual GNSS. However, for the single GNSS, there is a high probability that the satellites will be located far from the ZH placement, owing to the limited number of satellites. Therefore, it is necessary to analyze the ZH placement while considering a dual GNSS. From Table 2, it can be observed that, overall, when the dual GNSS satellite is selected, the HDOP features a strong correlation with the SST variable.

SST is the value expressed by Equation (15). This value is calculated solely from the uniform satellites of the individual GNSSs, as explained previously. As can be observed from Equation (13) and Table 2, the HDOP decreases as the SST decreases. If the satellites of each GNSS are placed uniformly, the two terms in Equation (15) approach 0, and the SST decreases. Therefore, to lower the HDOP, the satellites of individual GNSSs must be placed uniformly. This was confirmed through simulations involving five uniform satellites.

**Figure 3:**
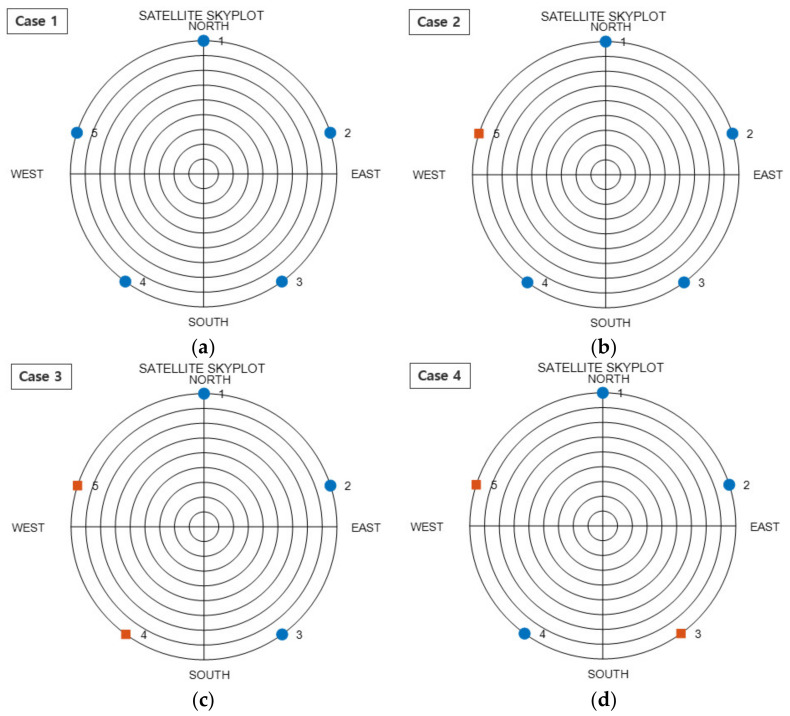
Squared Sum Trigonometric function (SST) simulation for five uniform satellites (blue-circle: first GNSS, red-square: second GNSS): (**a**) Case 1; (**b**) Case 2; (**c**) Case 3; (**d**) Case 4.

Using these five uniform satellites, four placements were created for a dual GNSS. These four placements are depicted in Figure 3. The SST values for the four cases are listed in Table 3. When the satellite placement was configured as a single GNSS, as in Case 1, the SST was 0. When one dual GNSS satellite was added, as in Case 2, the SST was 1. Further, when two dual GNSS satellites were added, as in Cases 3 and 4, the value varied according to the placement of satellites. As mentioned in the mathematical analysis above, the SST in Case 4, where the dual GNSS placement was arranged more uniformly, was lower than that in Case 3. In this manner, it can be observed that, as the satellites of individual GNSSs are placed more uniformly, the SST decreases; consequently, the HDOP decreases. This result holds even when the number of satellites increases.

### 5.2. Vertical Dilution of Precision

In the case of the VDOP, the simulations were performed for all of the cases with six selected satellites, without any additional conditions. The simulation results are presented in Table 4, in ascending order of the VDOP. Here, the table omits cases where the calculation was impossible owing to the insufficient rank of the H matrix. As described in Equation (14), it is difficult to identify a clear correlation between the VDOP and its variables. However, in the simulation results, tendencies can be determined in the satellite placements with low VDOP. The case with the lowest VDOP was the single GNSS case; this is similar to the HDOP results. The single GNSS had the minimum VDOP when the number of zenith satellites and the number of uniform satellites were equal, as in Equation (8). When a dual GNSS satellite was selected, the VDOP decreased as the number of zenith satellites and the number of uniform satellites within the individual GNSS became nearly equal.

Unlike the HDOP, the VDOP was simulated without additional conditions, and a relatively large number of simulations were required, as presented in Table 4. Thus, to facilitate the analyses, additional conditions were applied to the VDOP. These additional conditions were applied to the placement with the minimum VDOP, which consisted of three zenith and three uniform satellites. Table 5 lists the numbers of satellites for all possible cases, according to the type of dual GNSS in this scenario. These cases were applied when using actual satellites, as discussed in the following section.

## 6. Verification of Formulas Using Actual Satellites

**Figure 4:**
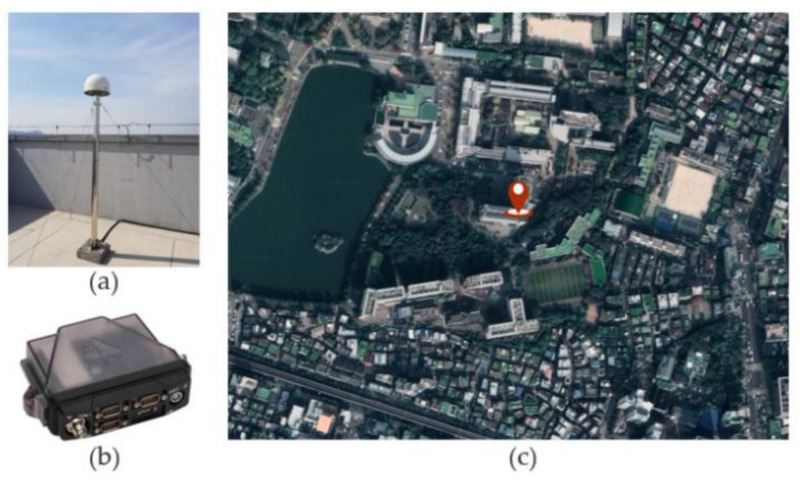
Actual data receiving environment: (**a**) Antenna (NOVATEL, GNSS-750); (**b**) Receiver (NOVTEL, OEM-6, Flexpak6); (**c**) Antenna Position.

The real-world data used in the analyses were acquired over the course of approximately 29 h, from 01:00 on 22 December 2021, to 06:00 on 23 December 2021. To avoid similarities in the satellite placement, sampling was performed at 15 min intervals. These data were acquired on the roof of the New Engineering Building of Konkuk University, Seoul, South Korea. A NOVATEL OEM6 was used as the receiver. The actual data receiving environment is given in Figure 4. GPS and Galileo were selected as the dual GNSSs, considering the number of visible satellites and the variety of satellite placements, from which the data were received.

In this study, four DOPs were calculated and compared at each time point. If the satellites used in the DOP calculations were from a single GNSS, the estimations were performed for a case involving four unknown values. Further, when dual GNSS satellites were selected, five unknown values were estimated. The first of the four DOPs was calculated using all visible satellites. For the second, the DOP values were calculated for all subsets, in which six satellites were selected from among all of the satellites, and the minimum DOP was used. The third DOP value was calculated by selecting the satellites closest to the ZH placement. Finally, the fourth DOP was calculated by changing the satellites that were selected in the third DOP, based on Section 4 and Section 5 considering a dual GNSS. These four DOPs are shown in following figure and table, and they are labeled as all-in-view (AIV), brute force (BF), regular polygon (RP), and case change (CC), respectively. These values were analyzed, and the previously described formulas were verified.

### 6.1. Horizontal Dilution of Precision

**Figure 5:**
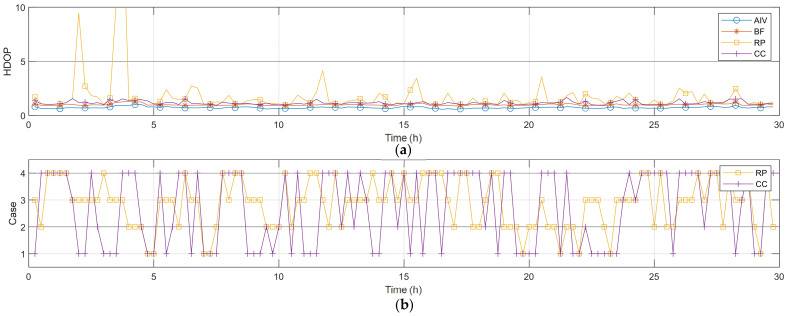
Actual data satellite selection horizontal results (**a**) HDOP; (**b**) uniform satellite case.

Figure 5 and Table 6 present the HDOP and statistical values obtained over time. AIV and BF naturally exhibited a low HDOP, and the average difference in the HDOP was approximately 0.27. By examining this difference, it is evident that when appropriate satellites are selected, there is no significant difference compared to the DOP determined using all satellites. In the case of RP, where the results were obtained considering satellites close to the ZH placement with one zenith satellite and five uniform satellites, high HDOP values were calculated at several time points. This is due to the fact that the closest satellites were selected, without considering the placement according to the type of GNSS. CC represents the results obtained by changing the uniform satellites in the RP placement in descending order of SST.

Algorithm 1 is the pseudo code of an algorithm for changing the uniform satellites. This algorithm was designed to change the satellites to those in Cases 3, 2, 4, and 1, which are arranged in descending order by SST. Only one satellite was changed at a time, except when Case 4 was changed to Case 1 at the end. Moreover, as the placements move farther from the ZH placement on changing the satellites, the HDOP values before and after the change were compared; notably, the change was not implemented if the HDOP value increased.
**Algorithm 1** Changing uniform satellite for HDOP improvement (n_u_ = 5)1: void function changing uniform satellite(azimuth_satellites, elevation_satellites)2:  n_u_ = 5;3:  sat_me = find satellite of minimum elevation(elevation_satellites);4:  vec_s = generate vector of satellites(azimuth_satellites, elevation_satellites);5:  vec_rp = generate vector of regular polygon(n_u_, sat_me);6:  sat_selec = select satellite of nearest regular polygon vector(vec_s, vec_rp);7:  case_selec = check case of selected satellite(sat_selec);8:  if case_selec == 39:   sat_case2 = change satellite from case 3 to case 2;10:   [case_selec, sat_selec] = compare case(case_selec, sat_selec, sat_case2);11:  end12:  if case_selec == 213:   sat_case1 = change satellite from case 2 to case 1;14:   sat_case4 = change satellite from case 2 to case 4;15:   [case_selec, sat_selec] = compare case(case_selec, sat_selec, sat_case1, sat_case4);16:  end17:  if case_selec == 418:   sat_case1 = change satellite from case 4 to case 1;19:   [case_selec, sat_selec] = compare case(case_selec, sat_selec, sat_case1);20:  end21:  return sat_selec, case_selec22: end function changing uniform satellite23:24: void function compare case(case_selec, sat_selec, sat_caseA, sat_caseB, …)25:  flag_case = compare minimum HDOP(sat_selec, sat_caseA, sat_caseB, …);26:  switch flag_case27:   case case_selec28:    return sat_selec, case_selec29:   case A30:    sat_selec = sat_caseA;31:    case_selec = A;32:    return sat_selec, case_selec33:   case B34:    …35:  end36: end function case selection

**Figure 6:**
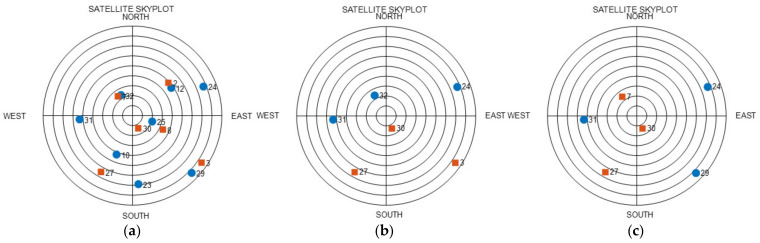
Skyplot, 3 h 45 min epoch (blue-circle: first GNSS, red-square: second GNSS): (**a**) AIV; (**b**) RP; (**c**) CC.

An analysis was performed at the time point with the highest HDOP in RP, as shown in Figure 5. The time point was 3 h and 45 min. A skyplot of this time is shown in Figure 6. The graph in Figure 6a is the skyplot of all visible satellites in the dual GNSS, and the HDOP at this time was calculated as 0.9331. As can be observed, there were no satellites in the northwestern sky at this time. The graph in Figure 6b shows the RP results, for which satellites close to the ZH placement were selected, and the HDOP was calculated as 20.0791. Satellite 30 in the second system was close to the zenith placement in the ZH placement, and the rest of the satellites were thus selected as the uniform satellites. With regard to the uniform satellites, three satellites from the first GNSS were selected in succession, and 2 satellites from the second GNSS were selected in succession, which represents the placement for Case 3 in Figure 3. Moreover, Case 3 entails the placement with the highest SST, and its HDOP was higher than that in the other cases. Furthermore, the satellites themselves were not visible in the northwestern sky, and satellites with low elevation angles could not be selected in that direction. Hence, it was predicted that a high HDOP can be calculated. The graph in Figure 6c shows the skyplot for CC, where the uniform satellites were changed using the algorithm, and the HDOP at this time was 1.5239. Owing to this change in the uniform satellites, the HDOP was reduced by 18.5552. The zenith satellites and satellites 24 and 31 in the first GNSS, as well as satellite 27 in the second GNSS, were not modified; the remaining two satellites were changed. The changed satellite positions were close to the RP satellites, and the type of GNSS was changed. This changed placement is depicted under Case 4 in Figure 3. Consequently, the HDOP was considerably reduced, solely on changing the type of GNSS in this manner. Based on these results, the previously presented conclusions were deemed to be valid.

Figure 5 indicates that, after applying the algorithm, the HDOP of CC was reduced in comparison to that for RP. Cases 2 and 3, which presented a high SST in the graph of Figure 5b and in Table 7, exhibited a reduction when moving from RP to CC. By contrast, Cases 1 and 4, which had a low SST, exhibited an increase. These results indicate that, Case change considering a dual GNSS proceeds for decreasing SST. This leads to a reduction in HDOP. These results demonstrate that it is important to perform satellite placement according to the dual GNSS.

### 6.2. Vertical Dilution of Precision

**Figure 7:**
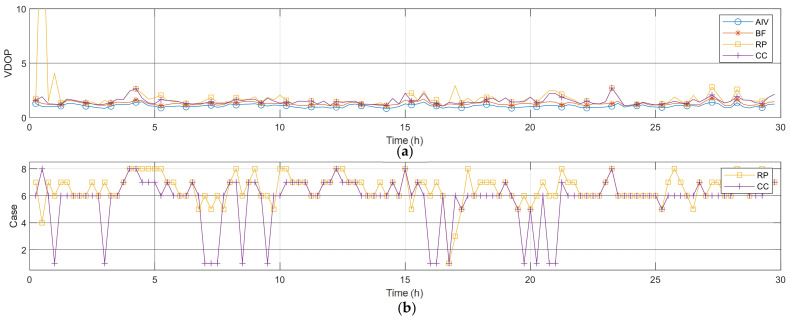
Actual data satellite selection vertical results (**a**) VDOP; (**b**) satellite case.

Figure 7 and Table 8 present the VDOP and statistical values over time. AIV and BF showed low VDOP values, and there was an average difference of approximately 0.22. For the VDOP, three zenith satellites and three uniform satellites were selected. Moreover, for the three zenith satellites in the ZH placement, three satellites with high elevation angles were selected. For the three uniform satellites, this study selected satellites that were closest to the triangular vectors created based on the lowest elevation angle. The VDOP calculated using the satellites selected in this manner is shown as RP. In these results as well, there were intervals where the VDOP increased. To compensate for this, a VDOP satellite placement changing algorithm was applied, as in the case of the HDOP.

Algorithm 2 the pseudo code of an algorithm for changing the VDOP satellite placement. Based on previous results, it can be predicted that the minimum VDOP in Table 5 is obtained under Case 1, whereas the maximum VDOP is obtained under Case 4. This algorithm changed one satellite at a time, starting with Case 4, which had the maximum VDOP. The interval within which the case change was performed included the process of comparing the VDOP before and after the satellite change and determining whether a change occurred.
**Algorithm 2** Changing satellite for VDOP improvement (*n* = 6)1: void function changing satellite(azimuth_satellites, elevation_satellites)2:  n_z_ = 3;3:  n_u_ = 3;4:  sat_me = find satellite of minimum elevation(elevation_satellites);5:  sat_zenith = select satellite of high elevation(n_z_, azimuth_satellites);6:  vec_s = generate vector of satellites(azimuth_satellites, elevation_satellites);7:  vec_rp = generate vector of regular polygon(n_u_, sat_me);8:  sat_uniform = select satellite of nearest regular polygon vector(vec_s, vec_rp);9:  sat_selec = merge zenith and uniform satellites(sat_zenith, sat_uniform);10:   case_selec = check case of selected satellite(sat_selec);11:   if case_selec == 412:    sat_case8 = change satellite from case 4 to case 8;13:    sat_case3 = change satellite from case 4 to case 3;14:    [case_selec, sat_selec] = compare case(case_selec, sat_selec, sat_case8, sat_case3);15:   end16:   if case_selec == 817:    sat_case7 = change satellite from case 8 to case 7;18:    [case_selec, sat_selec] = compare case(case_selec, sat_selec, sat_case7);19:   end20:   if case_selec == 321:    sat_case7 = change satellite from case 3 to case 7;22:    sat_case2 = change satellite from case 3 to case 2;23:    [case_selec, sat_selec] = compare case(case_selec, sat_selec, sat_case7, sat_case2);24:   end25:   if case_selec == 526:    sat_case6 = change satellite from case 5 to case 6;27:    [case_selec, sat_selec] = compare case(case_selec, sat_selec, sat_case6);28:   end29:   if case_selec == 730:    sat_case6 = change satellite from case 7 to case 6;31:    [case_selec, sat_selec] = compare case(case_selec, sat_selec, sat_case6);32:   end33:   if case_selec == 234:    sat_case6 = change satellite from case 2 to case 6;35:    [case_selec, sat_selec] = compare case(case_selec, sat_selec, sat_case6);36:   end37:   if case_selec == 638:    sat_case1 = change satellite from case 6 to case 1;39:    [case_selec, sat_selec] = compare case(case_selec, sat_selec, sat_case1);40:   end41:   return sat_selec, case_selec42:  end function changing satellite43:44:  void function compare case(case_selec, sat_selec, sat_caseA, sat_caseB, …)45:   flag_case = compare minimum VDOP(sat_selec, sat_caseA, sat_caseB, …);46:   switch flag_case47:    case case_selec48:     return sat_selec, case_selec49:    case A50:     sat_selec = sat_caseA;51:     case_selec = A;52:     return sat_selec, case_selec53:    case B54:     …55:   end56:  end function case selection

**Figure 8:**
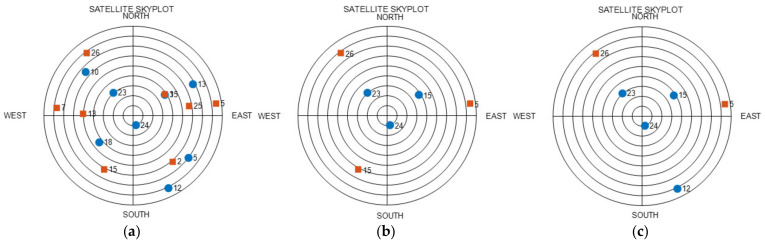
Skyplot, 30 min epoch (blue-circle: first GNSS, red-square: second GNSS): (**a**) AIV; (**b**) RP; (**c**) CC.

An analysis was performed at the time point where the largest values were calculated for the VDOP RP results. This time point was the 30 min point. The graph in Figure 8a is a skyplot of the AIV satellite, and the VDOP at this time was 1.0075. The graph in Figure 8b shows the RP results, and the VDOP at this time was 21.1959. The three zenith satellites in the satellite arrangement for this time were from the first GNSS, whereas the three uniform satellites were from the second GNSS. This arrangement is presented under Case 4 in Table 5. Case 4 represents the arrangement in which the VDOP is considered to be the highest; it can be observed that this analysis is consistent with the actual satellite selection. The graph in Figure 8c presents a skyplot for the results of changing the satellites, while considering a dual GNSS. Among the RP satellites, only Satellite 15 of the second GNSS was changed to Satellite 12 of the first GNSS. When such changes were implemented, the VDOP was 1.9062, which indicates a reduction of 19.2897 in comparison to that for RP. Thus, the VDOP was significantly reduced on changing the type of GNSS, although the satellites were farther away from the ZH placement after this change. These results indicate that the methods studied thus far are also valid in the case of the VDOP.

Table 9 presents the number of case selections before and after the VDOP satellite change. When the satellite changes were performed considering the GNSS, there was an increase in the numbers of selections for Cases 1 and 6, which exhibit low VDOP values, and a decrease in the numbers of selections for the other cases. With regard to the HDOP, the placement according to the dual GNSS type is also important for VDOP.

## 7. Conclusions

In this study, we mathematically derived the HDOP and VDOP for dual GNSS satellites in the ZH placement, which is an ideal condition, and verified these results through simulations and actual satellite data. For the ZH method used in previous studies, minimum GDOP conditions were confirmed through simulations. In this study, the ZH placement conditions were used without modifications, and the DOP was calculated mathematically. Furthermore, the HDOP and VDOP were differentiated and mathematically derived, rather than the GDOP. The results confirmed that the HDOP decreases as the number of uniform satellites in a single GNSS increases, whereas the VDOP decreases as the numbers of zenith and uniform satellites become more similar. This indicates that the DOP values vary according to the numbers of satellites placed at the zenith and those placed uniformly. Thus, satellite placements can be changed and used depending on the importance of horizontal or vertical placement for a given field of application.

There are two methods for handling the receiver clock errors in each system when using dual GNSSs. This study employed the method that handles errors by adding unknown values. The HDOP and VDOP for a dual GNSS in the ZH placement were mathematically derived, and simulations were performed. As in the case of a single GNSS, the HDOP for a dual GNSS decreases as the number of uniform satellites increases. Further, it was confirmed that the SST variable has a strong correlation with the HDOP for a dual GNSS. The SST is a variable that indicates the uniformity of the satellites in each individual GNSS of a dual GNSS. It was found that SST and HDOP decrease as the individual GNSS satellites’ uniformity increases. Based on the VDOP formula, it was difficult to confirm a clear correlation between the variables and the VDOP. One reason for this is that the numbers of zenith and uniform satellites both have the same correlation with VDOP, but the sum of the numbers of zenith and uniform satellites is constant, and they cannot both cause a reduction in the VDOP simultaneously. Therefore, the characteristics of the VDOP were confirmed through simulation results. In the simulation results, the VDOP decreased as an individual GNSS’ number of zenith satellites and the number of uniform satellites in the dual GNSS became nearly equal. In this manner, it was confirmed that the HDOP and VDOP tendencies of a single GNSS are also maintained in the dual GNSS. Therefore, it is predicted that these tendencies will be maintained even on expanding to multiple GNSS.

Through the simulation results, it was confirmed that the HDOP and VDOP of a single GNSS are lower than those of a dual GNSS. These results suggest that, if the ZH placement is to be implemented, it is more effective to use single GNSS satellites than dual GNSS satellites. However, this may differ when actual satellites that are closest to the ZH placement are selected, given that it is difficult for actual satellites to remain in the perfect ZH placement. In the case of a single GNSS, the number of satellites is small; therefore, it is more unlikely that the satellites are close to the ZH placement, as compared to those in the dual GNSS. Consequently, further research on satellite placement is necessary to lower the HDOP and VDOP for a dual GNSS. To confirm this, actual satellite data were used. When satellites close to the ZH placement were selected, without considering the type of GNSS, there were intervals within which the HDOP and VDOP both increased considerably. In this study, a satellite change algorithm that considers the type of GNSS was used. The results confirmed that the increase in the HDOP and VDOP was reduced. In addition, on applying this algorithm, the number of satellite placement cases for lower HDOP and VDOP values increases. These results confirmed that the research presented herein is valid.

It should be noted that this study was limited to investigating a dual GNSS owing to the mathematical complexity involved. Nevertheless, it was confirmed that the characteristics of a single GNSS are maintained even for a dual GNSS. Therefore, it can be expected that the same tendencies will also be maintained when multiple GNSSs are considered. In addition, since these results were derived mathematically, it is expected that the same tendencies will exist when selecting a larger number of satellites, as opposed to the limited number of satellites adopted in this study. Since this study aims to mathematically derive and verify DOP, we did not analyze the optimality of the satellite selection method in detail. However, it explained the necessity and effectiveness of satellite selection. Therefore, this study is expected to be applicable to various satellite selection methods.

## Figures and Tables

**Table 1:** Dilution of precision (DOP) simulation of single GNSS ZH satellite placement.

n	$n_{z}:n_{u}$	GDOP	HDOP	VDOP
4	1:3	1.7321	1.1547	1.1547
5	1:4	1.5811	1.0000	1.1180
5	2:3	1.5811	1.1547	0.9129
6	1:5	1.4832	0.8944	1.0954
6	2:4	1.4142	1.0000	0.8660
6	3:3	1.5275	1.1547	0.8165
7	1:6	1.4142	0.8165	1.0801
7	2:5	1.3038	0.8944	0.8367
7	3:4	1.3540	1.0000	0.7638
7	4:3	1.5000	1.1547	0.7638
8	1:7	1.3628	0.7559	1.0690
8	2:6	1.2247	0.8165	0.8165
8	3:5	1.2383	0.8944	0.7303
8	4:4	1.3229	1.0000	0.7071
8	5:3	1.4832	1.1547	0.7303

**Table 2:** Horizontal dilution of precision (HDOP) simulation (zenith: 1, uniform: 5).

$n_{z}^{a}$	$n_{z}^{b}$	$n_{u}^{a}$	$n_{u}^{b}$	nC	SST	HDOP
1	0	5	0	0	0.0000	0.8944
1	0	3	2	6	0.3820	0.9265
0	1	3	2	6	0.3820	0.9265
1	0	4	1	4	1.0000	1.0954
0	1	4	1	4	1.0000	1.0954
1	0	3	2	6	2.6180	1.8819
0	1	3	2	6	2.6180	1.8819
0	1	5	0	0	0.0000	X

**Table 3:** SST for satellite placement.

Case	1	2	3	4
SST	0.0000	1.0000	2.6180	0.3820

**Table 4:** Vertical dilution of precision (VDOP) simulation (*n* = 6).

$n_{z}^{a}$	$n_{z}^{b}$	$n_{u}^{a}$	$n_{u}^{b}$	nC	SST	VDOP
3	0	3	0	0	0.0000	0.8165
2	1	2	1	12	1.0000	0.8165
2	0	4	0	0	0.0000	0.8660
1	1	2	2	12	0.0000	0.8660
1	1	2	2	12	2.0000	0.8660
2	1	3	0	6	0.0000	0.9129
1	2	2	1	12	1.0000	0.9129
1	1	3	1	10	1.0000	0.9129
2	0	2	2	8	0.0000	1.0000
0	2	2	2	8	0.0000	1.0000
2	0	3	1	6	1.0000	1.0000
1	0	5	0	0	0.0000	1.0954
1	1	4	0	4	0.0000	1.1180
1	0	4	1	4	1.0000	1.1402
1	2	3	0	6	0.0000	1.1547
1	0	3	2	6	0.3820	1.1631
0	1	3	2	6	0.3820	1.2425
1	0	3	2	6	2.6180	1.4991
0	1	4	1	4	1.0000	1.6733
0	2	3	1	6	1.0000	1.7321

**Table 5:** VDOP case (zenith satellites: three, uniform satellites: three).

Case	Zenith (First GNSS: Second GNSS)	Uniform (First GNSS: Second GNSS)
1	3:0	3:0
2	3:0	2:1
3	3:0	1:2
4	3:0	0:3
5	2:1	3:0
6	2:1	2:1
7	2:1	1:2

**Table 6:** HDOP statistical results by type of satellite selection.

Selection	Min	Mean	Max
AIV	0.5689	0.7228	1.0050
BF	0.8780	0.9883	1.2848
RP	0.9668	1.7770	20.0791
CC	0.9418	1.1551	1.6639

**Table 7:** Number and percentage of HDOP cases after applying the algorithm.

Case	RP		CC	
	Number	Percentage	Number	Percentage
1	9	7.563	43	36.134
2	31	26.050	11	9.244
3	46	38.656	5	4.202
4	33	27.731	60	50.420
Sum	119	100.000	119	100.000

**Table 8:** VDOP statistical results by type of satellite selection.

	Min	Mean	Max
AIV	0.8371	1.0633	1.4352
BF	1.0457	1.2810	1.7934
RP	1.0457	1.8160	21.1959
CC	1.0457	1.5087	2.7137

**Table 9:** Number and percentage of VDOP cases after applying the algorithm.

Case	RP		CC	
	Number	Percentage	Number	Percentage
1	1	0.840	14	11.765
2	0	0.000	0	0.000
3	1	0.840	0	0.000
4	1	0.840	0	0.000
5	10	8.404	4	3.361
6	45	37.815	66	55.462
7	42	35.294	29	24.370
8	19	15.967	6	5.042
Sum	119	100.000	119	100.000

## Data Availability

Not applicable.

## Appendix A

(10)
$$\begin{array}{ll} H^{T}H & =\left[\begin{array}{ccccc} \cos^{2}\theta \sum_{k=1}^{n_{u}}\sin^{2}\phi^{k} & 0 & 0 & -\cos\theta \sum_{k=1}^{n_{u}^{a}}\sin\phi^{k} & -\cos\theta \sum_{k=1}^{n_{u}^{b}}\sin\phi^{k}\\ & \cos^{2}\theta \sum_{k=1}^{n_{u}}\cos^{2}\phi^{k} & 0 & -\cos\theta \sum_{k=1}^{n_{u}^{a}}\cos\phi^{k} & -\cos\theta \sum_{k=1}^{n_{u}^{b}}\cos\phi^{k}\\ & & n_{z}+n_{u}\sin^{2}\theta & -n_{z}^{a}-n_{u}^{a}\sin\theta & -n_{z}^{b}-n_{u}^{b}\sin\theta \\ & & & n^{a} & 0\\ & & & & n^{b} \end{array}\right]\\ \; & =\left[\begin{array}{ccccc} \frac{n_{u}}{2}\cos^{2}\theta & 0 & 0 & -SS\cos\theta & SS\cos\theta \\ & \frac{n_{u}}{2}\cos^{2}\theta & 0 & -SC\cos\theta & SC\cos\theta \\ & & n_{z}+n_{u}\sin^{2}\theta & -n_{z}^{a}-n_{u}^{a}\sin\theta & -n_{z}^{b}-n_{u}^{b}\sin\theta \\ & & & n^{a} & 0\\ & & & & n^{b} \end{array}\right]\\ \; & ∵\sum_{k=1}^{n_{u}^{a}}\sin\phi^{k}+\sum_{k=1}^{n_{u}^{b}}\sin\phi^{k}=0,\sum_{k=1}^{n_{u}^{a}}\cos\phi^{k}+\sum_{k=1}^{n_{u}^{b}}\cos\phi^{k}=0\\ \; & ∵SS:=\sum_{k=1}^{n_{u}^{a}}\sin\phi^{k},SC:=\sum_{k=1}^{n_{u}^{a}}\cos\phi^{k}\\ \; & ∵\sum_{k=1}^{n_{u}}\sin^{2}\phi^{k}=\sum_{k=1}^{n_{u}}\frac{1-\cos2\phi^{k}}{2}=\frac{n_{u}-\sum_{k=1}^{n_{u}}\cos2\phi^{k}}{2}=\frac{n_{u}}{2}\;\left(if\;n_{u}\geq 3,\;\sum_{k=1}^{n_{u}}\cos2\phi^{k}=0\right)\\ \; & ∵\sum_{k=1}^{n_{u}}\cos^{2}\phi^{k}=\sum_{k=1}^{n_{u}}\frac{1+\cos2\phi^{k}}{2}=\frac{n_{u}+\sum_{k=1}^{n_{u}}\cos2\phi^{k}}{2}=\frac{n_{u}}{2}\;\left(if\;n_{u}\geq 3,\;\sum_{k=1}^{n_{u}}\cos2\phi^{k}=0\right) \end{array}$$

(12)
$${\left(H^{T}H\right)}^{-1}=\left[\begin{array}{cc} {\left(B_{3\times 3}^{ENU}-B_{3\times 2}^{PC}{\left(B_{2\times 2}^{C}\right)}^{-1}{\left(B_{3\times 2}^{PC}\right)}^{T}\right)}^{-1} & \\ & {\left(B_{2\times 2}^{C}-{\left(B_{3\times 2}^{PC}\right)}^{T}{\left(B_{3\times 3}^{ENU}\right)}^{-1}B_{3\times 2}^{PC}\right)}^{-1} \end{array}\right]$$

(A1)
$$\begin{array}{cc} B_{3\times 2}^{PC}{\left(B_{2\times 2}^{C}\right)}^{-1}{\left(B_{3\times 2}^{PC}\right)}^{T} & =\left[\begin{array}{cc} -SS\cos\theta & SS\cos\theta \\ -SC\cos\theta & SC\cos\theta \\ -n_{z}^{a}-n_{u}^{a}\sin\theta & -n_{z}^{b}-n_{u}^{b}\sin\theta \end{array}\right]\left[\begin{array}{cc} 1/n^{a} & 0\\ 0 & 1/n^{b} \end{array}\right]\left[\begin{array}{ccc} -SS\cos\theta & -SC\cos\theta & -n_{z}^{a}-n_{u}^{a}\sin\theta \\ SS\cos\theta & SC\cos\theta & -n_{z}^{b}-n_{u}^{b}\sin\theta \end{array}\right]\\ \; & =\left[\begin{array}{cc} -\frac{1}{n^{a}}SS\cos\theta & \frac{1}{n^{b}}SS\cos\theta \\ -\frac{1}{n^{a}}SC\cos\theta & \frac{1}{n^{b}}SC\cos\theta \\ -\frac{n_{z}^{a}}{n^{a}}-\frac{n_{u}^{a}}{n^{a}}\sin\theta & -\frac{n_{z}^{b}}{n^{b}}-\frac{n_{u}^{b}}{n^{b}}\sin\theta \end{array}\right]\left[\begin{array}{ccc} -SS\cos\theta & -SC\cos\theta & -n_{z}^{a}-n_{u}^{a}\sin\theta \\ SS\cos\theta & SC\cos\theta & -n_{z}^{b}-n_{u}^{b}\sin\theta \end{array}\right]\\ \; & =\left[\begin{array}{ccc} \frac{n^{a}+n^{b}}{n^{a}n^{b}}SS^{2}\cos^{2}\theta & \frac{n^{a}+n^{b}}{n^{a}n^{b}}SS\cdot SC\cos^{2}\theta & \frac{\left(n_{u}^{a}n^{b}-n_{z}^{b}n^{a}\right)\sin\theta +n_{z}^{a}n^{b}-n_{z}^{b}n^{a}}{n^{a}n^{b}}SS\cos\theta \\ & \frac{n^{a}+n^{b}}{n^{a}n^{b}}SC^{2}\cos^{2}\theta & \frac{\left(n_{u}^{a}n^{b}-n_{z}^{b}n^{a}\right)\sin\theta +n_{z}^{a}n^{b}-n_{z}^{b}n^{a}}{n^{a}n^{b}}SC\cos\theta \\ & & \frac{n_{u}^{a}n_{u}^{a}n^{b}+n^{a}n_{u}^{b}n_{u}^{b}}{n^{a}n^{b}}\sin^{2}\theta +\frac{2\left(n_{z}^{a}n_{u}^{a}n^{b}+n^{a}n_{z}^{b}n_{u}^{b}\right)}{n^{a}n^{b}}\sin\theta +\frac{n_{z}^{a}n_{z}^{a}n^{b}+n^{a}n_{z}^{b}n_{z}^{b}}{n^{a}n^{b}} \end{array}\right] \end{array}$$

(A2)
$$\begin{array}{cc} B_{3\times 3}^{ENU}-B_{3\times 2}^{PC}{\left(B_{2\times 2}^{C}\right)}^{-1}{\left(B_{3\times 2}^{PC}\right)}^{T} & =\left[\begin{array}{ccc} h_{11} & h_{12} & h_{13}\\ & h_{22} & h_{23}\\ & & h_{33} \end{array}\right]\\ \; & =\left[\begin{array}{ccc} \frac{n_{u}}{2}\cos^{2}\theta -\frac{n^{a}+n^{b}}{n^{a}n^{b}}SS^{2}\cos^{2}\theta & -\frac{n^{a}+n^{b}}{n^{a}n^{b}}SS\cdot SC\cos^{2}\theta & -\frac{\left(n_{u}^{a}n^{b}-n_{z}^{b}n^{a}\right)\sin\theta +n_{z}^{a}n^{b}-n_{z}^{b}n^{a}}{n^{a}n^{b}}SS\cos\theta \\ & \frac{n_{u}}{2}\cos^{2}\theta -\frac{n^{a}+n^{b}}{n^{a}n^{b}}SC^{2}\cos^{2}\theta & -\frac{\left(n_{u}^{a}n^{b}-n_{z}^{b}n^{a}\right)\sin\theta +n_{z}^{a}n^{b}-n_{z}^{b}n^{a}}{n^{a}n^{b}}SC\cos\theta \\ & & n_{z}+n_{u}\sin^{2}\theta -\frac{n_{u}^{a}n_{u}^{a}n^{b}+n^{a}n_{u}^{b}n_{u}^{b}}{n^{a}n^{b}}\sin^{2}\theta -\frac{2\left(n_{z}^{a}n_{u}^{a}n^{b}+n^{a}n_{z}^{b}n_{u}^{b}\right)}{n^{a}n^{b}}\sin\theta -\frac{n_{z}^{a}n_{z}^{a}n^{b}+n^{a}n_{z}^{b}n_{z}^{b}}{n^{a}n^{b}} \end{array}\right]\\ \; & =\left[\begin{array}{ccc} \left(\frac{n_{u}}{2}-\frac{n^{a}+n^{b}}{n^{a}n^{b}}SS^{2}\right)\cos^{2}\theta & \frac{n^{a}+n^{b}}{n^{a}n^{b}}SS\cdot SC\cos^{2}\theta & \frac{n_{u}^{a}n_{z}^{b}-n_{z}^{a}n_{u}^{b}}{n^{a}n^{b}}\left(\sin\theta -1\right)SS\cos\theta \\ & \left(\frac{n_{u}}{2}-\frac{n^{a}+n^{b}}{n^{a}n^{b}}SC^{2}\right)\cos^{2}\theta & \frac{n_{u}^{a}n_{z}^{b}-n_{z}^{a}n_{u}^{b}}{n^{a}n^{b}}\left(\sin\theta -1\right)SC\cos\theta \\ & & \frac{n_{z}^{a}n_{u}^{a}n_{u}^{b}+n_{z}^{a}n_{u}^{a}n_{z}^{b}+n_{u}^{a}n_{z}^{b}n_{u}^{b}+n_{z}^{a}n_{z}^{b}n_{u}^{b}}{n^{a}n^{b}}{\left(\sin\theta -1\right)}^{2} \end{array}\right]\\ \; & ∵n^{a}=n_{z}^{a}+n_{u}^{a},\;n^{b}=n_{z}^{b}+n_{u}^{b},\;n_{u}=n_{u}^{a}+n_{u}^{b},\;n_{z}=n_{z}^{a}+n_{z}^{b} \end{array}$$

(A3)
$$\begin{array}{cc} \det\left(B_{3\times 3}^{ENU}-B_{3\times 2}^{PC}{\left(B_{2\times 2}^{C}\right)}^{-1}{\left(B_{3\times 2}^{PC}\right)}^{T}\right) & =h_{11}h_{22}h_{33}-h_{11}h_{23}^{2}-h_{12}^{2}h_{33}-h_{13}^{2}h_{22}+2h_{12}h_{13}h_{23}\\ & =\left(\left(\frac{n_{u}^{2}\left(n_{z}^{a}n_{u}^{a}n_{u}^{b}+n_{z}^{a}n_{u}^{a}n_{z}^{b}+n_{u}^{a}n_{z}^{b}n_{u}^{b}+n_{z}^{a}n_{z}^{b}n_{u}^{b}\right)}{4n^{a}n^{b}}\right)-\frac{{\left(n_{u}^{a}+n_{u}^{b}\right)}^{2}\left(n_{z}^{a}+n_{z}^{b}\right)}{2n^{a}n^{b}}\left(SS^{2}+SC^{2}\right)\right)\cos^{4}\theta {\left(\sin\theta -1\right)}^{2} \end{array}$$

(A3.1)
$$\begin{array}{cc} h_{11}h_{22}h_{33} & =\left(\left(\frac{n_{u}}{2}-\frac{n^{a}+n^{b}}{n^{a}n^{b}}SS^{2}\right)\cos^{2}\theta \right)\left(\left(\frac{n_{u}}{2}-\frac{n^{a}+n^{b}}{n^{a}n^{b}}SC^{2}\right)\cos^{2}\theta \right)\left(\frac{n_{z}^{a}n_{u}^{a}n_{u}^{b}+n_{z}^{a}n_{u}^{a}n_{z}^{b}+n_{u}^{a}n_{z}^{b}n_{u}^{b}+n_{z}^{a}n_{z}^{b}n_{u}^{b}}{n^{a}n^{b}}{\left(\sin\theta -1\right)}^{2}\right)\\ \; & =\left(\frac{n}{n^{a}n^{b}}SS^{2}-\frac{n_{u}}{2}\right)\left(\frac{n}{n^{a}n^{b}}SC^{2}-\frac{n_{u}}{2}\right)\left(\frac{n_{z}^{a}n_{u}^{a}n_{u}^{b}+n_{z}^{a}n_{u}^{a}n_{z}^{b}+n_{u}^{a}n_{z}^{b}n_{u}^{b}+n_{z}^{a}n_{z}^{b}n_{u}^{b}}{n^{a}n^{b}}\right)\cos^{4}\theta {\left(\sin\theta -1\right)}^{2} \end{array}$$

(A3.2)
$$\begin{array}{cc} h_{11}h_{23}^{2} & =\left(\left(\frac{n_{u}}{2}-\frac{n^{a}+n^{b}}{n^{a}n^{b}}SS^{2}\right)\cos^{2}\theta \right){\left(\frac{n_{u}^{a}n_{z}^{b}-n_{z}^{a}n_{u}^{b}}{n^{a}n^{b}}\left(\sin\theta -1\right)SC\cos\theta \right)}^{2}\\ & ={\left(\frac{n_{u}^{a}n_{z}^{b}-n_{z}^{a}n_{u}^{b}}{n^{a}n^{b}}\right)}^{2}\left(\frac{n_{u}}{2}-\frac{n}{n^{a}n^{b}}SS^{2}\right)SC^{2}\cos^{4}\theta {\left(\sin\theta -1\right)}^{2} \end{array}$$

(A3.3)
$$\begin{array}{cc} h_{12}^{2}h_{33} & ={\left(\frac{n^{a}+n^{b}}{n^{a}n^{b}}SS\cdot SC\cos^{2}\theta \right)}^{2}\left(\frac{n_{z}^{a}n_{u}^{a}n_{u}^{b}+n_{z}^{a}n_{u}^{a}n_{z}^{b}+n_{u}^{a}n_{z}^{b}n_{u}^{b}+n_{z}^{a}n_{z}^{b}n_{u}^{b}}{n^{a}n^{b}}{\left(\sin\theta -1\right)}^{2}\right)\\ & ={\left(\frac{n}{n^{a}n^{b}}\right)}^{2}SS^{2}\cdot SC^{2}\frac{n_{z}^{a}n_{u}^{a}n_{u}^{b}+n_{z}^{a}n_{u}^{a}n_{z}^{b}+n_{u}^{a}n_{z}^{b}n_{u}^{b}+n_{z}^{a}n_{z}^{b}n_{u}^{b}}{n^{a}n^{b}}{\left(\sin\theta -1\right)}^{2}\cos^{4}\theta \end{array}$$

(A3.4)
$$\begin{array}{cc} h_{13}^{2}h_{22} & ={\left(\frac{n_{u}^{a}n_{z}^{b}-n_{z}^{a}n_{u}^{b}}{n^{a}n^{b}}\left(\sin\theta -1\right)SS\cos\theta \right)}^{2}\left(\left(\frac{n_{u}}{2}-\frac{n^{a}+n^{b}}{n^{a}n^{b}}SC^{2}\right)\cos^{2}\theta \right)\\ & ={\left(\frac{n_{u}^{a}n_{z}^{b}-n_{z}^{a}n_{u}^{b}}{n^{a}n^{b}}\right)}^{2}\left(\frac{n_{u}}{2}-\frac{n}{n^{a}n^{b}}SC^{2}\right)SS^{2}{\left(\sin\theta -1\right)}^{2}\cos^{4}\theta \end{array}$$

(A3.5)
$$\begin{array}{cc} 2h_{12}h_{13}h_{23} & =2\left(\frac{n^{a}+n^{b}}{n^{a}n^{b}}SS\cdot SC\cos^{2}\theta \right)\left(\frac{n_{u}^{a}n_{z}^{b}-n_{z}^{a}n_{u}^{b}}{n^{a}n^{b}}\left(\sin\theta -1\right)SS\cos\theta \right)\left(\frac{n_{u}^{a}n_{z}^{b}-n_{z}^{a}n_{u}^{b}}{n^{a}n^{b}}\left(\sin\theta -1\right)SC\cos\theta \right)\\ & =2\left(\frac{n}{n^{a}n^{b}}\right){\left(\frac{n_{u}^{a}n_{z}^{b}-n_{z}^{a}n_{u}^{b}}{n^{a}n^{b}}\right)}^{2}SS^{2}\cdot SC^{2}\cos^{4}\theta {\left(\sin\theta -1\right)}^{2} \end{array}$$

(A4)
$$\begin{array}{cc} HDOP^{2} & =\frac{h_{22}h_{33}-h_{23}^{2}+h_{11}h_{33}-h_{13}^{2}}{\det\left(B_{3\times 3}^{ENU}-B_{3\times 2}^{PC}{\left(B_{2\times 2}^{C}\right)}^{-1}{\left(B_{3\times 2}^{PC}\right)}^{T}\right)}=\frac{h_{33}\left(h_{11}+h_{22}\right)-\left(h_{13}^{2}+h_{23}^{2}\right)}{\det\left(B_{3\times 3}^{ENU}-B_{3\times 2}^{PC}{\left(B_{2\times 2}^{C}\right)}^{-1}{\left(B_{3\times 2}^{PC}\right)}^{T}\right)}\\ \; & =\frac{4}{n_{u}\cos^{2}\theta }\frac{\left(n_{z}^{a}n_{u}^{a}n_{u}^{b}+n_{z}^{a}n_{u}^{a}n_{z}^{b}+n_{u}^{a}n_{z}^{b}n_{u}^{b}+n_{z}^{a}n_{z}^{b}n_{u}^{b}\right)-n_{z}\left(SS^{2}+SC^{2}\right)}{\left(n_{z}^{a}n_{u}^{a}n_{u}^{b}+n_{z}^{a}n_{u}^{a}n_{z}^{b}+n_{u}^{a}n_{z}^{b}n_{u}^{b}+n_{z}^{a}n_{z}^{b}n_{u}^{b}\right)-2n_{z}\left(SS^{2}+SC^{2}\right)} \end{array}$$

(A4.1)
$$\begin{array}{cc} h_{33}\left(h_{11}+h_{22}\right) & =\frac{n_{z}^{a}n_{u}^{a}n_{u}^{b}+n_{z}^{a}n_{u}^{a}n_{z}^{b}+n_{u}^{a}n_{z}^{b}n_{u}^{b}+n_{z}^{a}n_{z}^{b}n_{u}^{b}}{n^{a}n^{b}}{\left(\sin\theta -1\right)}^{2}\left(\left(\frac{n_{u}}{2}-\frac{n^{a}+n^{b}}{n^{a}n^{b}}SS^{2}\right)\cos^{2}\theta +\left(\frac{n_{u}}{2}-\frac{n^{a}+n^{b}}{n^{a}n^{b}}SC^{2}\right)\cos^{2}\theta \right)\\ & =\left(n_{u}-\frac{n^{a}+n^{b}}{n^{a}n^{b}}\left(SS^{2}+SC^{2}\right)\right)\frac{n_{z}^{a}n_{u}^{a}n_{u}^{b}+n_{z}^{a}n_{u}^{a}n_{z}^{b}+n_{u}^{a}n_{z}^{b}n_{u}^{b}+n_{z}^{a}n_{z}^{b}n_{u}^{b}}{n^{a}n^{b}}\cos^{2}\theta {\left(\sin\theta -1\right)}^{2} \end{array}$$

(A4.2)
$$\begin{array}{cc} h_{13}^{2}+h_{23}^{2} & ={\left(\frac{n_{u}^{a}n_{z}^{b}-n_{z}^{a}n_{u}^{b}}{n^{a}n^{b}}\left(\sin\theta -1\right)SS\cos\theta \right)}^{2}+{\left(\frac{n_{u}^{a}n_{z}^{b}-n_{z}^{a}n_{u}^{b}}{n^{a}n^{b}}\left(\sin\theta -1\right)SC\cos\theta \right)}^{2}\\ & ={\left(\frac{n_{u}^{a}n_{z}^{b}-n_{z}^{a}n_{u}^{b}}{n^{a}n^{b}}\right)}^{2}\left(SS^{2}+SC^{2}\right){\left(\sin\theta -1\right)}^{2}\cos^{2}\theta \end{array}$$

(A5)
$$\begin{array}{cc} VDOP^{2} & =\frac{h_{11}h_{22}-h_{12}^{2}}{\det\left(B_{3\times 3}^{ENU}-B_{3\times 2}^{PC}{\left(B_{2\times 2}^{C}\right)}^{-1}{\left(B_{3\times 2}^{PC}\right)}^{T}\right)}\\ & =\frac{n^{a}n^{b}-\frac{2n}{n_{u}}\left(SS^{2}+SC^{2}\right)}{\left(n_{z}^{a}n_{u}^{a}n_{u}^{b}+n_{z}^{a}n_{u}^{a}n_{z}^{b}+n_{u}^{a}n_{z}^{b}n_{u}^{b}+n_{z}^{a}n_{z}^{b}n_{u}^{b}\right)-2n_{z}\left(SS^{2}+SC^{2}\right)}\frac{1}{{\left(1-\sin\theta \right)}^{2}} \end{array}$$

(A5.1)
$$\begin{array}{cc} h_{11}h_{22}-h_{12}^{2} & =\left(\frac{n_{u}}{2}-\frac{n^{a}+n^{b}}{n^{a}n^{b}}SS^{2}\right)\cos^{2}\theta \left(\frac{n_{u}}{2}-\frac{n^{a}+n^{b}}{n^{a}n^{b}}SC^{2}\right)\cos^{2}\theta -{\left(\frac{n^{a}+n^{b}}{n^{a}n^{b}}SS\cdot SC\cos^{2}\theta \right)}^{2}\\ & =\left({\left(\frac{n_{u}}{2}\right)}^{2}-\frac{n_{u}n}{2n^{a}n^{b}}\right)\cos^{4}\theta \end{array}$$
